# Identifying the causal allele in the CD40 autoimmune locus enables discovery of context-specific trans-effects in B cells

**DOI:** 10.64898/2026.07.29.741564

**Published:** 2026-08-04

**Authors:** Yoshihiko Tomofuji, Zepeng Mu, Hafsa Mire, Yu Zhao, Cassidy Liu, Vidya Jayanthi, Nicholas Sugiarto, Accelerating Medicines Partnership®: RA/SLE Network, Jeffrey A. Sparks, Soumya Raychaudhuri

## Abstract

Thousands of genetic variants are associated with autoimmune diseases, but causal variants, their mechanisms, and the pathogenic context in which they act are elusive. Knowledge of pathogenic contexts may enable effective targeted therapies, instead of broad immunosuppressive approaches. First, to focus on the genetics of immune response, we used surface marker CITE-seq data from 1,055,857 peripheral blood mononuclear cells from 356 individuals. We defined genetic associations to 148 surface proteins across eight cell types. We observed a signal in the CD40 locus, implicated in rheumatoid arthritis (RA) and other autoimmune conditions. RA risk variants increased CD40 protein expression by ∼20% on B cells, but with minimal mRNA effects. Second, we deployed base-resolution genome editing, with CRAFT-seq, capturing genomic DNA sequence at the edited site and multimodal phenotypes at single-cell resolution. We defined a single causal allele, rs1883832, within the Kozak motif. Third, we edited this allele, in primary B cells and conducted CRAFTseq to demonstrate *trans* -effects in >200 genes. These effects were only in the light zone germinal center-like state. Importantly, these *trans* -effects were not seen in population-scale cohorts of unstimulated B cells. This represents a framework to define disease causal alleles, their *cis* – and *trans* -effects. It demonstrates the power of defining causal genetic variation to find *trans* -effects through editing, which cannot easily be found in population studies.

## Full Text Availability

The license terms selected by the author(s) for this preprint version do not permit archiving in PMC. The full text is available from the preprint server.

